# Whole-Brain Reconstruction of Neurons in the Ventral Pallidum Reveals Diverse Projection Patterns

**DOI:** 10.3389/fnana.2021.801354

**Published:** 2021-12-16

**Authors:** Qiru Feng, Sile An, Ruiyu Wang, Rui Lin, Anan Li, Hui Gong, Minmin Luo

**Affiliations:** ^1^School of Life Science, Tsinghua University, Beijing, China; ^2^Peking University - Tsinghua University-National Institute Biological Science (PTN) Joint Graduate Program, School of Life Science, Tsinghua University, Beijing, China; ^3^National Institute of Biological Science, Beijing, China; ^4^Wuhan National Laboratory for Optoelectronics, Ministry of Education Key Laboratory for Biomedical Photonics, Britton Chance Center for Biomedical Photonics, Huazhong University of Science and Technology, Wuhan, China; ^5^School of Life Science, Peking University, Beijing, China; ^6^Huazhong University of Science and Technology (HUST)-Suzhou Institute for Brainsmatics, Jiangsu Industrial Technology Research Institute (JITRI), Suzhou, China; ^7^Tsinghua Institute of Multidisciplinary Biomedical Research, Beijing, China; ^8^Chinese Institute for Brain Research, Beijing, China

**Keywords:** neuronal morphology, sparse labeling, fMOST, single neuron reconstruction, whole-brain mapping, reward

## Abstract

The ventral pallidum (VP) integrates reward signals to regulate cognitive, emotional, and motor processes associated with motivational salience. Previous studies have revealed that the VP projects axons to many cortical and subcortical structures. However, descriptions of the neuronal morphologies and projection patterns of the VP neurons at the single neuron level are lacking, thus hindering the understanding of the wiring diagram of the VP. In this study, we used recently developed progress in robust sparse labeling and fluorescence micro-optical sectioning tomography imaging system (fMOST) to label mediodorsal thalamus-projecting neurons in the VP and obtain high-resolution whole-brain imaging data. Based on these data, we reconstructed VP neurons and classified them into three types according to their fiber projection patterns. We systematically compared the axonal density in various downstream centers and analyzed the soma distribution and dendritic morphologies of the various subtypes at the single neuron level. Our study thus provides a detailed characterization of the morphological features of VP neurons, laying a foundation for exploring the neural circuit organization underlying the important behavioral functions of VP.

## Introduction

The ventral pallidum (VP), a major structure of the basal ganglia, represents a central station in the brain reward circuits (Heimer et al., 1982; Zahm, 1989). It integrates the reward signals mainly from the nucleus accumbens and regulates the execution of motivated behavior (Smith et al., 2009; Root et al., 2015; Richard et al., 2016), such as pleasure (Berridge, 2003; Smith and Berridge, 2005; Ahrens et al., 2016) and drug-seeking (Tang et al., 2005; Mahler et al., 2014). Bulk axonal tracing studies demonstrate that VP neurons directly project to the mediodorsal (MD) thalamus, lateral habenula (LHb), lateral hypothalamus (LHA), ventral tegmental area (VTA), and pedunculopontine tegmental nucleus (Groenewegen et al., 1993; Mahler et al., 2014; Leung and Balleine, 2015; Root et al., 2015; Knowland et al., 2017; Faget et al., 2018). Interestingly, the VP is heterogeneous in its constituent cell types, each of which may exert different functions. For example, a recent study reveals that GABAergic and glutamatergic neurons cells in the VP drive the opposite approach and avoidance behaviors (Heinsbroek et al., 2020; Stephenson-Jones et al., 2020).

The knowledge of the precise wiring diagrams of the VP is important for a full understanding of how VP neurons process information at both the local and global scales. Several projects have generated mesoscale connectomes for adult mouse brains, including the Allen Mouse Brain Connectivity Atlas (Oh et al., 2014) and the Mouse Brain Architecture Project (http://mouse.branarchitechture.org/). These resources have revealed connectivity matrices for various brain regions, including the VP. However, the overall projections of a cluster of neurons in the VP could not provide the detailed projections of individual neurons due to the labeling of a large number of intermixing neurons. In recent years, with the development of sparse labeling methods (Rotolo et al., 2008; Lin et al., 2018) and brain-wide precision imaging (Economo et al., 2016; Gong et al., 2016), more research attention has been focused on the whole-brain reconstruction of individual neurons (Peng et al., 2015; Economo et al., 2016; Li et al., 2018; Winnubst et al., 2019), including cortical neurons (Economo et al., 2016; Guo et al., 2017); cholinergic neurons (Li et al., 2018); dopamine neurons (Lin et al., 2018); serotonin neurons (Ren et al., 2019); and striatal, thalamic, cortical, and claustral neurons (Han, 2020). These results demonstrate that a neuron population within a brain area often exhibits multiple patterns of axonal projection at the single-cell level, which suggests a more precise circuit organization underlying the functional roles of a given neuron population.

Given the central role of the VP in behavioral motivation and the potential value of knowing VP wiring diagram at the single-cell level, in this study, we combined a sparse labeling technique (Lin et al., 2018) and the fluorescence micro-optical sectioning tomography (fMOST) technology (Gong et al., 2016) to reconstruct MD-projecting VP neurons at the whole-brain level. Detailed analyses of 41 fully reconstructed neurons allowed us to cluster VP neurons into three major subtypes, each of which displays distinct axonal projection patterns. This study is the first to reveal the complete morphology and distinct diversity projections of single neurons in the VP. Our results intend to provide a foundation for understanding the relationship between neuronal morphologies and behavioral functions of the VP.

## Materials and Methods

### Animals

Animal care and use were approved by the institutional guidelines of the National Institute of Biological Sciences, Beijing, and conformed to the regulations by the Administration of Affairs Concerning Experimental Animals of China. Adult (8–12 weeks old) C57BL/6N wild-type mice of either sex were used. Mice were maintained with a 12 h light/dark photoperiod. Food and water were provided *ad libitum*.

### Adeno-Associated Virus and Constructs Packaging

AAV2-retro-Cre was purchased from Shanghai Taitool Bioscience (Shanghai, China). We used the sparse labeling constructs pAAV-TRE-DIO-FLPo and pAAV-TRE-fDIO-GFP-IRES-tTA (Lin et al., 2018). AAV vectors were packaged into the AAV2/9 serotype. These two vectors and the AAV helper plasmids were amplified using the Stbl3 cell line, to reduce potential spontaneous recombination during bacterial growth. AAV viral vectors were packaged as described previously (Grieger et al., 2006). Briefly, AAV plasmids were co-transfected into HEK293T, cells were collected 72 h after transfection, and the viral particles were released from cells by freeze-thaw cycle and sonication. The viral vectors were purified and concentrated by cesium chloride density-gradient ultracentrifugation, dialyzed into HN buffer (20 mM HEPES, 145 mM sodium chloride, pH 7.8). The viral titer was identified using quantitative PCR; for FLPo (5′ to 3′), the forward primer is AGACCCTGTACCAGTTCCTG and the reverse primer is CGCTGAAAAAGTAGATGTGCC; and for GFP-IRES-tTA (5′ to 3′), the forward primer is ATTTTCCAGGGTTTCGTACTG and the reverse primer is GCATCATACCCACTTCTGC. Viruses were typically obtained at a titer of 1–5 10^9^ VG/μl.

### Surgery and Virus Injection

Mice were anesthetized using 1.25% v/v avertin (125–250 mg/kg body weight IP) and placed in a mouse stereotaxic instrument. The skin was cut, and a craniotomy was made. We injected 80 nl of AAV-retro-Cre (titer 5.6 × 109 VG/μl) into the MD (bregma −1.45 mm, lateral 0.5 mm, depth 3 mm). To achieve sparse labeling, the AAV-TRE-DIO-FLPo virus was diluted at a 1:100 ratio in HN buffer and combined at a 1:9 ratio with the AAV-TRE-fDIO-GFP-ires-tTA virus. A total of 60 nl of the AAV mixture was injected into the VP (bregma +0.3 mm, lateral 1.55 mm, depth 4.65 mm). Viruses were injected using a microsyringe pump (Nanoliter 2010 Injector, WPIf, Sarasota, FL, USA). A Micro4 controller was used to control the virus injection at the speed of 46 nl/min.

### fMOST Instrument

We used the fMOST system as described previously (Gong et al., 2016; Sun et al., 2019). Briefly, the fMOST system used a mercury lamp (X-Cite exacte, Lumen Dynamics, ON, Canada) as a light source, a digital micromirror device (DMD, XD-ED01N, X-Digit, Shanghai, China) for generating lighting grid patterns, and water-immersion objective (1.0NA, XLUMPLFLN 20XW, Olympus, Tokyo, Japan) for imaging. Two scientific complementary metal-oxide-semiconductor cameras (ORCA-Flash 4.0 and Hamamatsu Photonics K.K., Hamamatsu, Japan) were used for signal detection. Piezoelectric translational stage (P-725 PIFOC LongTravel Objective Scanner, E-753 Digital Piezo Controller, PI GmbH, Karlsruhe, Germany) moved the objective lens for axial scanning. The sample box is mounted to a high-precision 3D translation stage (ABL20020-ANT130-AVL125, Aerotech Inc., Pittsburgh, PA, USA). The 3D translation stage moved the sample for mosaic scanning and sectioning. A diamond knife (Diatome AG, Nidau, Switzerland) was used for sample sectioning.

### Sample Preparation and fmost Imaging

Mice were anesthetized with an overdose of pentobarbital, intracardially perfused with 0.9% saline and then 4% paraformaldehyde in 0.01 M phosphate-buffered saline (PBS; 1×). The brain was postfixed in 4% paraformaldehyde for 24 h at 4°C. The brain sample was washed in 1× PBS three times at an interval of 3 h and then subsequently dehydrated *via* immersion in a series of ethanol mixture solutions. Following dehydration, the brain was embedded in Lowicryl HM20 resin (Warrington, PA, USA) (Ren et al., 2018). The resin-embedded sample was fixed in a bath containing propidium iodide (PI) under the fMOST system at a voxel resolution of 0.23 × 0.23 × 1 μm. The samples in a coronal plane were imaged in two channels: the green channel was used to obtain the signal of labeled neurons and the red channel was used to obtain the cellular architecture information of PI. The entire mouse brain produced a 15–20 TB dataset containing about 10,000 coronal planes.

### Image Preprocessing and Neuron Reconstruction

Raw data preprocessing was used according to published methods (Ding et al., 2013; Gong et al., 2016). The preprocessing included image registration, illumination correction, and noise reduction. The images of Green Fluorescent Protein (GFP) and PI channels were compressed to 16-bit and 8-bit in Tiff format. We used Amira software with TDat plugin (Thermo Fisher Amira, Pittsburgh, PA, USA) (Li et al., 2017) to reconstruct neural skeletons. Briefly, we first identified a soma as the seed point and then navigated through the image stacks to connect the signal node to the seed point. To improve the efficiency of reconstruction, we chose semiautomatic tracing at sparsely distributed long-distance fibers. The linear tracing was used for complex terminal arborizations. Finally, the reconstruction neurons were subjected to quality control. The loops within neural skeletons were checked with built-in functions to eliminate erroneous crossing points and thus ensured that all reconstructed neurons maintain tree structure before registration and quantitative analysis.

We then registered the neuronal morphological data to the Allen Mouse Brain Common Coordinate Framework (CCF version 3, Allen Institute for Brain Science, Seattle, WA). The PI channel was first resampled at a resolution of 10 × 10 × 10 μm, which allowed us to use cytoarchitectures to sufficiently distinguish brain regions. We used a robust Brain Spatial Mapping Interface (BrainsMapi) to achieve accurate 3D registration of the data brains to the reference brain (Ni et al., 2020).

### Data Analysis

We used customized MATLAB programs to calculate basic morphological features, including fiber length, branch points, Sholl analysis, soma distribution, and labeling intensity in different brain regions. Hierarchical clustering was used for fine-level classification of neuron subclasses. The brain regions used are enlisted in Supplementary Table 1. Graphpad Prism (Madison, WI, USA) was used for statistical tests and plots. Exact *P*-values and corresponding statistic methods are shown in figures.

## Results

### Sparse Labeling and 3D Reconstruction of VP Projection Neurons

**Figure 1:**
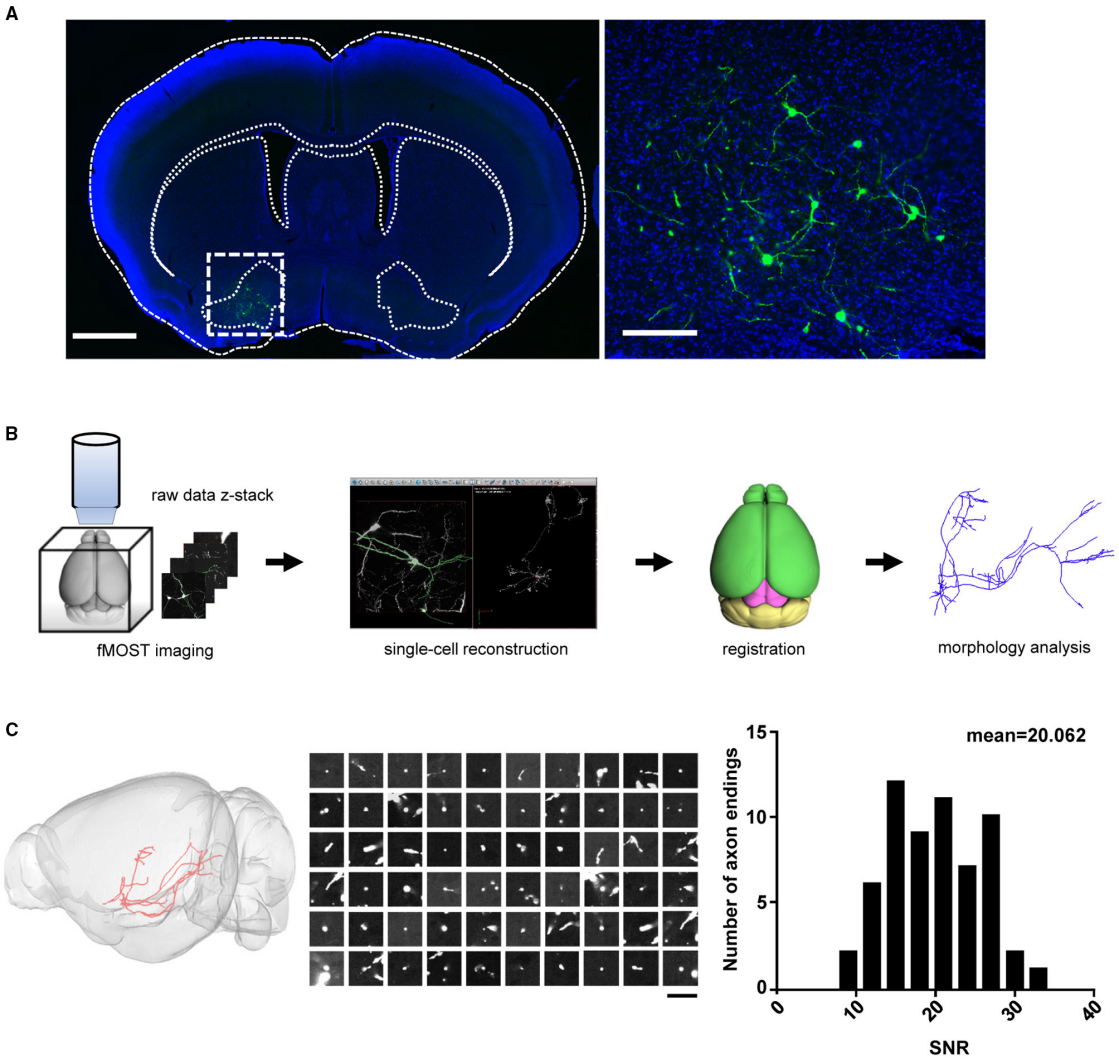
Sparse labeling and whole-brain reconstruction of the VP neurons. **(A)** GFP expression (green) following the infusion of AAV-Cre into the MD and the Cre-dependent dual-AAV vectors into the VP. Left: coronal view of the brain section containing the VP (scale bar = 1 mm). Right: zoom-in view of the dashed-box area in left (scale bar = 200 μm). **(B)** Workflow of imaging and whole-brain reconstruction of individual neurons. **(C)** Distribution of SNR for the axon endings. The left panel shows the labeling of a single VP projection neuron. The middle panel shows the raw images of 60 randomly chosen axonal terminal endings for the SNR calculation (scale bar = 20 μm). The SNR indicates the mean signal divided by the standard deviation of the background. The right panel shows the distribution histogram of the SNR values (*n* = 60 axonal endings). The high SNR values indicated an abrupt ending that is consistent with strong terminal labeling. VP, ventral pallidum; MD, mediodorsal nucleus of thalamus; SNR, signal-to-noise ratio.

Whole-brain reconstruction of VP projection neurons requires intense and sparse labeling to the full extent of distal axonal terminals. This had been challenging because the VP has a rather irregular shape, and there has been no specific driver animal line (Root et al., 2015). To overcome this challenge, we used previous findings that a majority of VP neurons project to the MD nucleus and its nearby nuclei in the thalamus (Zahm et al., 1996; Root et al., 2015) and that the AAV-retro vector allows efficient retrograde transportation of AAV vectors for driving gene expression (Tervo, 2016). We thus infused the AAV-retro-Cre virus into the MD and its adjacent areas of C57BL/6N mouse brains and the Cre-dependent dual AAV-vectors (AAV-TRE-DIO-FLPo; 10^7^ VG/μl and AAV-fDIO-GFP-ires-tTA; 10^9^ VG/μl) into the VP for sparse labeling of VP projection neurons (Supplementary Figure 1A). The expression of the Cre recombinase and the leaky expression of FLPo in VP neurons triggered positive feedback expression of tTA-TRE and GFP in a bicistronic manner (Lin et al., 2018) (Supplementary Figure 1B). Three weeks after AAV vector infusions, we observed strong GFP labeling in a sparse set of neurons within the VP (Figure 1A).

**Figure 2:**
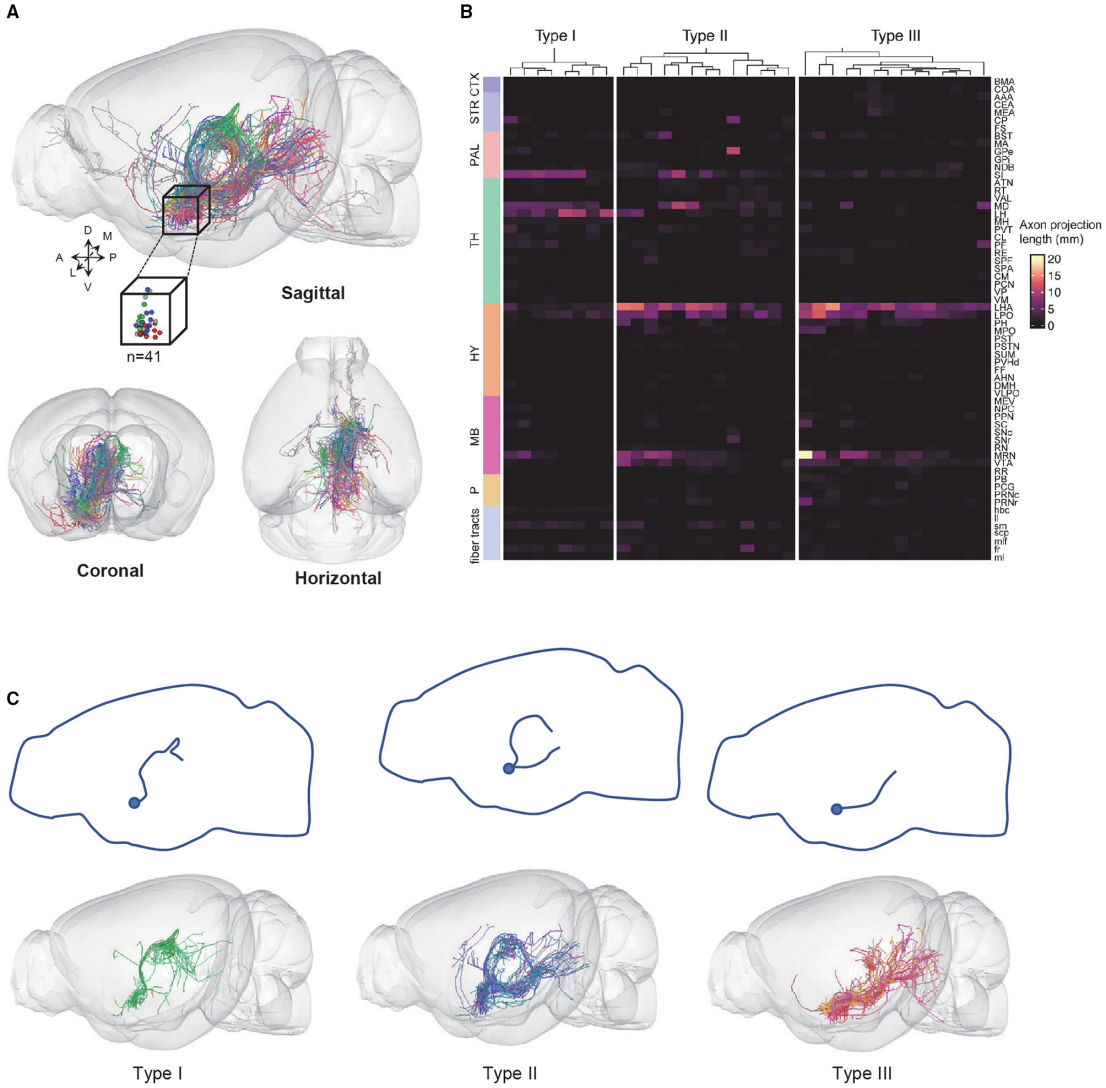
VP neurons are classified into three types based on axonal projection patterns. **(A)** 3D views of 41 fully reconstructed VP neurons (top: sagittal view with the cube representing the somatic location; bottom: coronal and horizontal views). **(B)** The axonal fiber density of 3 VP neurons types in the entire brain. Columns represent individual neurons and rows represent brain regions; fiber tracts. **(C)** The projection routes of the three types of VP neurons. The top panels show a projection route schematic diagram. The bottom panels show the overlapped views of the individual VP neurons. CTX, cerebral cortex; STR, striatum; PAL, pallidum; TH, thalamus; HY, hypothalamus; MB, midbrain; P, pons.

To obtain high-resolution imaging of the entire mouse brain, we embedded brain samples in resin and conducted imaging and raw data acquisition using the fMOST system (Gong et al., 2016) (Figure 1B). For each brain, we obtained an image set of ~6,000 image stacks at a voxel resolution of 0.23 × 0.23 × 1 μm in a coronal plane. The image data contain two imaging channels: one shows the GFP-positive neurons and the other shows cell bodies counterstained with PI. The PI channel was used to map the boundaries between brain regions by comparing differences in cell architecture (Hezel et al., 2012). The TB-size datasets were transformed into the TDat platform (Li et al., 2017) for digital reconstruction using the Amira software. High-resolution fMOST images allowed us to visualize somata and local dendrites, as well as the fine structures of distal axonal terminals. We examined each cell endpoint and analyzed the signal-to-noise ratio (SNR) of cross-sections containing a range of axonal morphologies, to ensure the accurate morphology and fiber terminal integrity of the reconstructed neurons (Figure 1C). Using the Amira software, we successfully reconstructed a total of 41 neurons from two mouse brains and then performed data registration to the Allen Mouse Brain Common Coordinate Framework (Figure 2A; Supplementary Figure 2 shows each neuron in 3D view). Consistent with previous tracing studies and the Allen mesoscale connectivity database (Groenewegen et al., 1993; Bell et al., 1995; Root et al., 2015), the axonal fibers from VP neurons were densely distributed in several brain regions, such as the LHb, MD, lateral preoptic area (LPO), LHA, VTA, and midbrain (MB) reticular nucleus (MRN), as well as in several major fiber tracts, such as the fasciculus retroflexus (fr), medial lemniscus (ml), and rubrospinal tract (rust) (Figure 2A). We then carried out detailed and quantitative analyses of the complete morphology and projection pattern of individual neurons rather than the overall projections of the VP neuron population.

### Classification of the VP Neurons and Quantitative Analysis of Axonal Projections

Out of the 41 neurons, 35 neurons could be classified into three subtypes based on the clustering of terminal distribution patterns in 64 brain areas that exhibited at least some labeling of axonal terminals: 8 type I neurons, 13 type II neurons, and 14 type III neurons (Figure 2B). Six neurons did not belong to these three types (“undefined”) and were thus not further analyzed. Interestingly, the three subtypes exhibited distinct projection routes. All type I neurons, whose axons extended from the anterior of the dorsal thalamus to bilateral MD and LHb that were bilaterally connected by the habenular commissure (hbc) (Figure 2C, left). The type II neurons contained two major projection routes, i.e., the one extending from the paraventricular nucleus of the thalamus (PVT) to the MB and the other extending from the lateral hypothalamic area to the pons (Figure 2C, middle). The type III neurons extended from the LHA through the parafascicular nucleus (PF) to the MD, MRN, and pons, respectively (Figure 2C, right).

**Figure 3:**
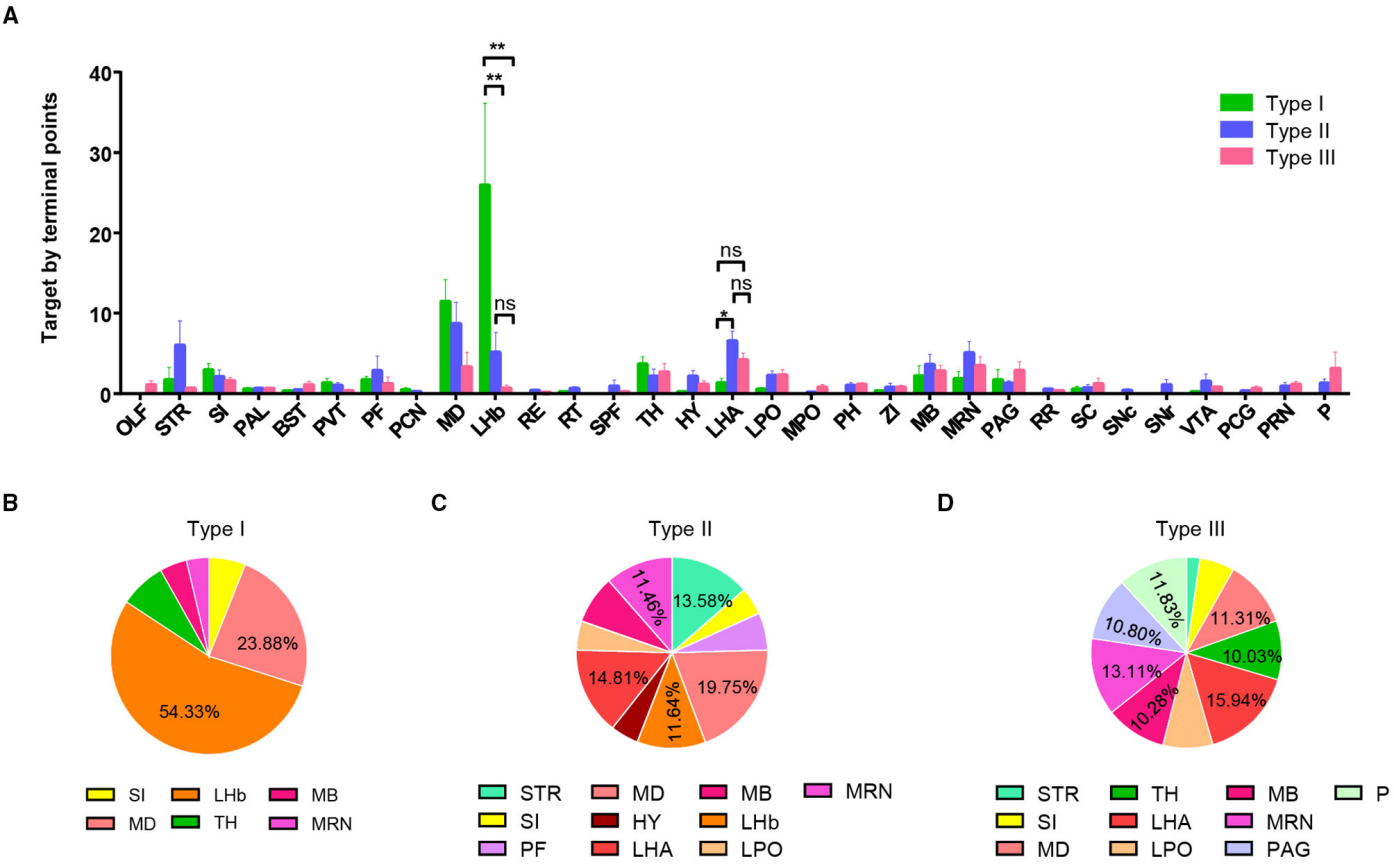
Quantification of projection of VP neurons in the whole brain. **(A)** Projection intensity of three types of VP neurons in the whole brain (**P < 0.01). **(B–D)** Pie charts showing a preference of projection intensity pattern in the whole brain for type I neurons **(B)**, type II neurons **(C)**, and type III neurons **(D)**. ns, no significant correlations; OLF, olfactory area; SI, subtantia innominate; BST, bed nuclei of the stria terminalis; PVT, paraventricular nucleus of the thalamus; PF, parafascicular nucleus; PCN, paracentral nucleus; LHb, lateral habenula; RE, nucleus of reuniens; RT, reticular nucleus of the thalamus; SPF, subparafascicular nucleus; LHA, lateral hypothalamic area; LPO, lateral preoptic area; MPO, medial preoptic area; PH, posterior hypothalamic nucleus; ZI, zona incerta; MRN, midbrain reticular nucleus; PAG, periaqueductal gray; RR, MB reticular nucleus, retrorubral area; SC, superior colliculus; SNc, substania nigra, compact part; SNr, substantia nigra, reticular part; VTA, ventral tegmental nucleus; PCG, pontine central gray; PRN, pontine reticular nucleus.

To further understand each type of projection characteristic, we used the number of axon terminal points as the indicator of projection strength in a specific brain area and performed a statistical analysis of projection strength in the three types of neurons. We selected brain regions with a projection strength average >1.5 (at least more than one branch) and calculated the percentage of these brain regions (Figure 3A). The results display different projection preferences of the three types. Type I neurons are mainly projected to the LHb (54.3%) and MD (23.4%) (Figure 3B). Type II neurons projected to five major areas including the MD (19.8%), LHA (14.8%), LHb (11.6%), STR (striatum, 13.6%), and MRN (MB reticular nucleus, 11.5%) (Figure 3C). Type III neurons widely and evenly projected to multiple targets, including the TH (thalamus, 10.0%), MD (11.3%), LHA (15.9%), MRN (10.3%), PAG (periaqueductal gray, 10.8%), and pons (11.8%) (Figure 3D).

**Figure 4:**
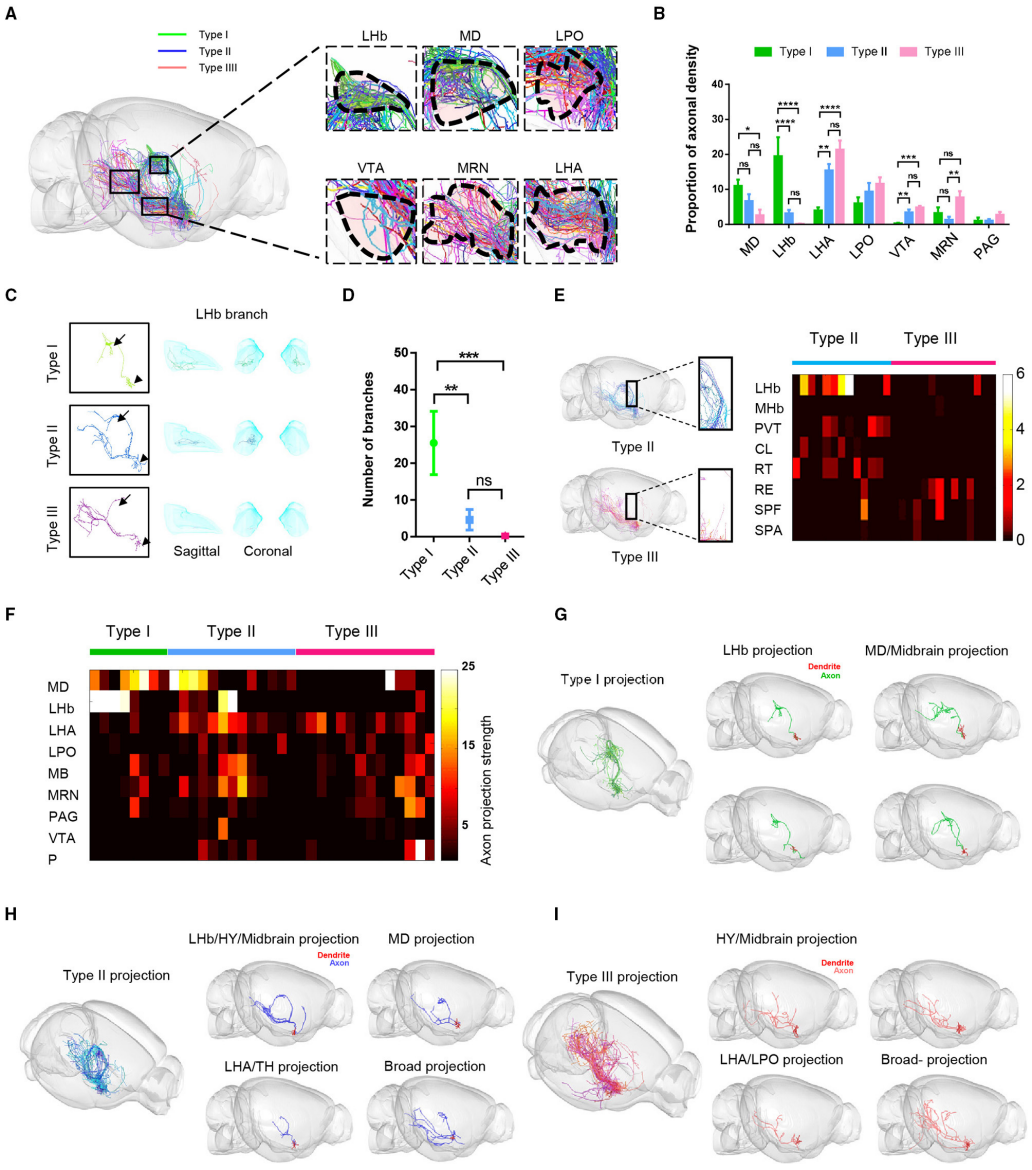
Fiber distribution patterns of the three types of VP neurons. **(A)** The sagittal view of the color-coded fiber distribution of VP neuron types. **(B)** Quantification analysis of the percentage of axon density in **(A)**. The three types show the difference in MD, LHb, LHA, VTA, and MRN; **P < 0.0067, ***P = 0.0001, ****P < 0.0001, one-way ANOVA with Turkey's multiple comparisons test. **(C)** Morphology of a representative type I, type II, and type III neuron. Arrowheads point to the somata and the arrows to the LHb. **(D)** The number of bifurcation points for the three types of VP neurons in the LHb. **P = 0.0026, ***P = 0.0002; *t*-tests; error bar indicates SEM. **(E)** Axonal fiber distribution of type II and type III neurons in the thalamus. The boxed areas in the left show zoom-in views of axonal segments in the PVT and RT. The heatmap on the right shows axonal labeling intensity. **(F)** The heatmap shows the number of end-terminal points in various brain regions. Rows: brain regions, columns: individual neurons. **(G–I)** Representative structural images show morphological heterogeneity within type I **(G)**, type II **(H)**, and type III neurons **(I)**. CL, central lateral nucleus of the thalamus; SPA, subparafascicular area.

To further describe the characteristics of long-range axon distribution of the three types of VP neurons, we quantitatively analyzed and compared the axonal labeling intensity in the key downstream areas including the MD, LHb, LHA, LPO, MRN, VTA, and PAG. We observed significant differences among the three cell types (Figures 4A,B). Type I VP neurons projected dense axonal terminals in the MD and the LHb. In contrast, type II and type III axons were more densely distributed in the LHA, LPO, and VTA than type I. This difference in labeling intensity was also associated with axonal branching points. For example, the number of axonal branches of type I neurons was significantly higher than those of type II and type III neurons within the LHb, thus suggesting that type I neurons had more extensive axonal ramifications and stronger synaptic output in the LHb than type II and type III neurons (Figures 4C,D). Although type II neurons and type III neurons exhibited rather similar long-range axon distribution patterns in the hypothalamus (HY) and MB, type II neurons exhibited overall stronger axonal projections in several thalamic nuclei, including the PVT, central lateral nucleus of the thalamus (CL), and reticular nucleus of the thalamus (RT), whereas type III neurons tended to project more heavily to the nucleus of reuniens (RE) within the MB thalamus as well as the MRN and PAG (Figures 4B,E).

In addition, we found even for these neurons belonging to the same category with similar projection routes, their projection patterns could exhibit some subtle differences. We drew a heatmap of the axonal densities within major projection brain regions of the whole brain at the single-cell level (Figure 4F). These data showed the diversity of VP neurons projections within the same category. For example, type I neurons could be divided into two subgroups: one subgroup of neurons mainly projected to bilateral LHb (*n* = 4 of 8 cells) and the other subgroup neurons intensively projected to the MD and MB (*n* = 4 of 8 cells) (Figures 4F,G). Similarly, some individual type II neurons projected more heavily to the MD (*n* = 4 of 13 cells), whereas some exhibited preference in their projections to the LHb/HY/MB (*n* = 2 of 13 cells), to the LHA/TH (*n* = 5 of 13 cells), or rather much more broadly (*n* = 2 of 13 cells) (Figures 4F,H). Finally, some type III neurons exhibited projection preference in the LHA/LPO (*n* = 7 of 14 cells), some in the HY/MB (*n* = 6 of 14 cells), and one rather broadly (Figure 4I).

### The Soma and Dendritic Morphology of the Three Types of VP Neurons

**Figure 5:**
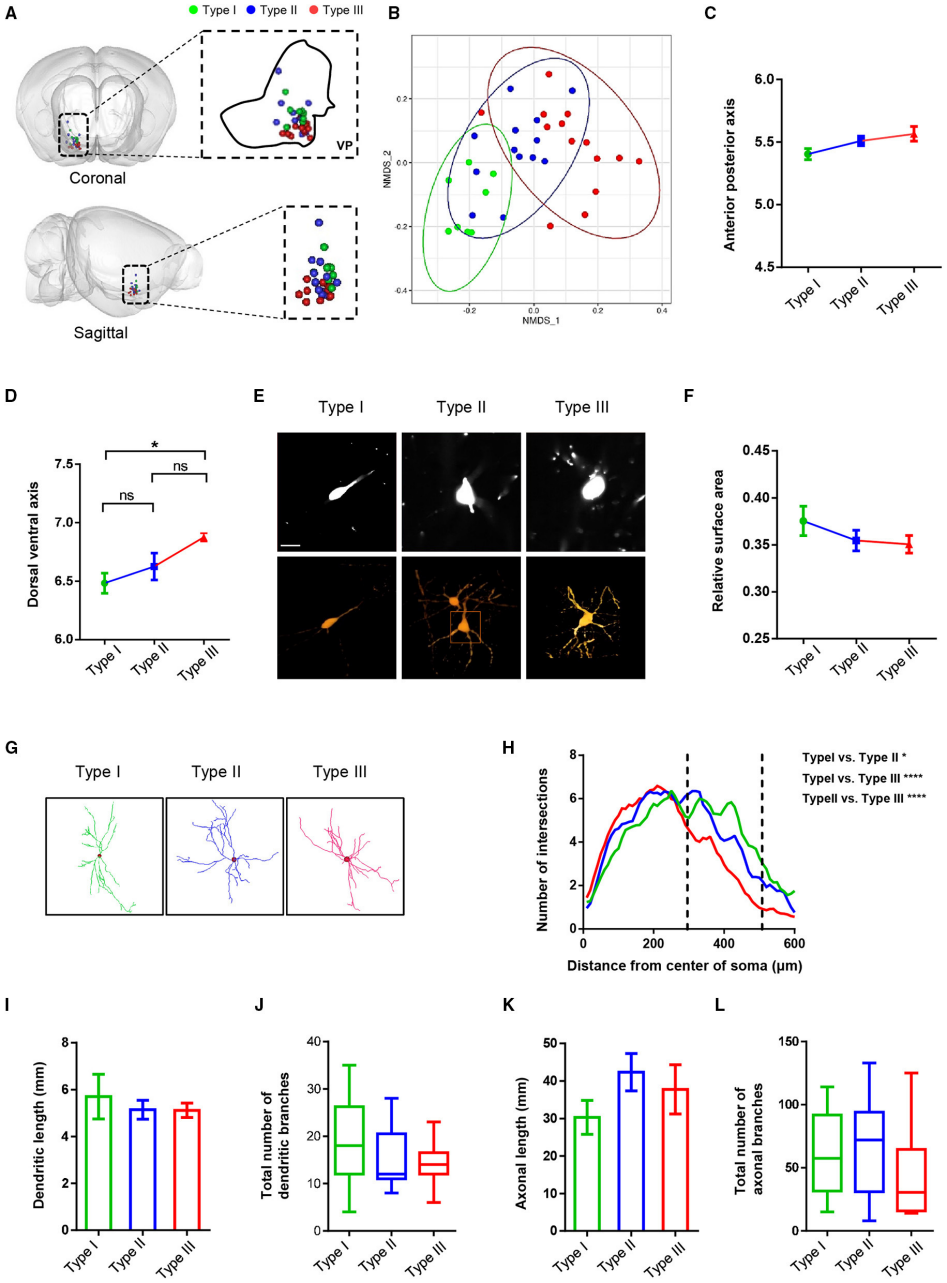
The morphological characteristics of VP neurons at the level of somata and dendrites. **(A)** Cell body position distribution of reconstructed neurons in the VP (the green blot is type I, the blue blot is type II, and the red blot is type III; top, coronal view; bottom, sagittal view). **(B)** Non-metric multidimensional scaling (NMDS) plot of cell body clusters. **(C,D)** Statistical comparisons of the cell body location along the anterior-posterior axis **(C)** and the dorsal-ventral axis **(D)**. The error bars indicate SEM. **(E)** Example of somatic morphology of three VP neurons (upper: fMOST image of somata; scale bar = 30 μm; lower, texture-based volume rendering image of the somata). **(F)** Comparison of surface area to volume ratio of reconstruction neurons. The error bars indicate SEM. **(G)** Images show the dendritic morphology of representative type I, type II, and type III neurons. **(H)** Sholl analysis of dendritic arborization. *P = 0.043, ****P < 0.0001; two-way ANOVA. **(I–L)** Comparison of dendritic length **(I)**, the total number of dendritic branches **(J)**, axonal length **(K)**, and the total number of axonal branches **(L)** of three types of neurons in the VP. The error bars indicate SEM.

In addition to the axonal projection patterns, we analyzed and compared a series of morphological characteristics, such as soma position, dendritic morphology, dendritic length, and overall axonal length and branches (Figure 5). Dimension reduction analysis of the 3D coordinates for the VP neuron somata indicated that the somata spatial location of reconstructed VP neurons showed a nonrandom distribution (Figures 5A,B). More specifically, along the anterior-posterior (AP) axis, the soma position of type I was more anterior than those of type II and type III neurons. Along the dorsal-ventral (DV) axis, the soma position of type III neurons was significantly ventral to those of type I and type II neurons (Figures 5C,D).

We also measured and compared the relative surface areas (surface area to volume ratio) of the three types of cell bodies. The result showed a tendency of the type I cell body to be smaller than type II and type III and no significant difference between type II and type III (Figures 5E,F). Sholl analysis of the dendritic morphology of the three types of neurons revealed that type III neurons exhibited fewer branches in distal dendrites than type I and type II neurons, although the overall dendrite lengths and the number of dendritic branches were similar across the three types of VP neurons (Figures 5G–J). Finally, all three types of VP neurons exhibited similar overall axon length and number of axonal branches (Figures 5K,L).

## Discussion

Brain-wide, cellular-level, mesoscale connectomes provide valuable information about the organization of neural circuits and contribute to the understanding of neural computation (Oh et al., 2014; Peng et al., 2015; Yuan et al., 2015). Owing to variations in the projections of individual neurons, visualizing the complete morphologies of neurons at a single cell level rather than obtaining projections of a cluster of neurons is important for further understanding the wiring diagram of the nervous system (Economo et al., 2016, 2019; Guo et al., 2017; Zhang et al., 2017; Sun et al., 2019; Winnubst et al., 2019). The VP is considered a key node in the brain reward system, but all existing mesoscale connectivity of the VP neurons only shows the overall projections of a large number of neurons (Zahm, 1989; Bell et al., 1995; Mahler et al., 2014; Root et al., 2015). In this study, we combined sparse labeling technology (Lin et al., 2018) and the fMOST system (Gong et al., 2016) to obtain high-resolution imaging data to reconstruct VP neurons at the whole-brain level. Our results reveal that VP neurons can be classified into at least three major subtypes with distinct axonal projection patterns and dendritic morphologies, suggesting functional heterogeneity among individual VP neurons.

Our labeling and imaging approaches enabled continuous series of high-resolution imaging and thus complete brain-wide reconstruction of 41 individual neurons. Our data provide detailed information on not only dendritic morphology but also the distal axonal projection patterns. This overcomes the limits of bolus tracer injections, which label a large number of neurons and dense axonal fibers and obscure the details of individual axonal organizations (Groenewegen et al., 1993; Mahler et al., 2014; Leung and Balleine, 2015; Faget et al., 2018; Heinsbroek et al., 2020; Stephenson-Jones et al., 2020; Pribiag et al., 2021). The 41 VP neurons were divided into three types according to the axonal projection patterns of individual neurons. All three neuron types project to the MD, which is believed to be important for executive functions, such as cognitive control and working memory (Napier and Mitrovic, 1999; Ferry et al., 2000; Kalivas et al., 2001; Leung and Balleine, 2015; Saga et al., 2017; Faget et al., 2018). Type I neurons project most strongly to the LHb, which mainly processes negative reward signals and mediates decision-making regarding aversive stimuli (Knowland et al., 2017; Faget et al., 2018; Yang et al., 2018). Type II neurons and type III neurons are featured with strong outputs to the LHA and the LPO in the hypothalamus and the VTA in the MB, which promote motivational behavior (Smith et al., 2009; Tachibana and Hikosaka, 2012; Castro et al., 2015) and various forms of reward-seeking (McFarland and Kalivas, 2001; Perry and McNally, 2013; Root et al., 2013; Mahler et al., 2014). Compared with type III neurons, type II neurons project more heavily to several midline thalamic nuclei that are often associated with arousal (Li et al., 2020). By contrast, type III neurons have strong output to the MB MRN and PAG areas, which are involved in locomotor control associated with behavioral motivation (Johnson et al., 1996). Within the subtypes, we also observed some subtle heterogeneity. Therefore, the distinct axonal projection patterns suggest that different VP neuron types channel outputs to mediate various aspects of reward-related behavior.

We find that the somata of the three VP neuron types tend to cluster in different subregions in the VP. Previous studies of VP anatomy have reported that the VP comprises the medial, ventromedial, and dorsal lateral subregions that correspond to VPm, VPvm, and VPdl. These subregions function in the regulation of different behavioral circuits. For example, the VPm receive projection from the nucleus accumbens shell (NAcSh) and project to the LHA to regulate food intake. The VPvm and VPdl receive projections from the medial shell and the core of the NAc, respectively, then project to the LHA, MD, VTA, and substantia nigra (SNr), and take part in processing natural rewards, controlling working memory, and regulating drugs of abuse behavior (Zahm and Heimer, 1990; Stratford et al., 1999; Stratford and Wirtshafter, 2012; Root et al., 2013, 2015; Faget et al., 2018). It requires further investigation to determine how the cell body position information we provided relates to these known neural circuits.

In this study, we focused on reconstructing VP neurons by driving the expression of fluorescent protein in neurons that project to the thalamic MD nucleus, which has been considered the key target of the VP projection (Zahm et al., 1996; Root et al., 2015). This approach allowed us to overcome the limitations associated with the lack of a specific driver mouse line for the VP. It is possible that some VP neurons do not project to the MD, suggesting the need for more comprehensive targeting to fully understand the connectivity of individual VP neurons. The VP consists of neurons of GABAergic, glutamatergic, and cholinergic phenotypes. Indeed, both GABAergic neurons and glutamatergic neurons in the VP project to the LHb and mediate opposite behaviors (Faget et al., 2018; Stephenson-Jones et al., 2020). Future studies that label VP neurons in a neurotransmitter-specific manner might enable us to analyze the morphological features associated with different neurotransmitter phenotypes.

In general, we obtained a continuous and complete morphology of neurons in the VP based on our whole-brain 3D data and analyzed the projections of these neurons. As far as we know, this study is the first to report a comprehensive morphological analysis of VP neurons with high spatial resolution. Our data will be helpful to deepen the understanding of the wiring diagram of the VP at the single neuron level and provide a foundation for exploring the behavioral functions of the VP-related neural circuits.

## Data Availability Statement

The original contributions presented in the study are included in the article/Supplementary Material, further inquiries can be directed to the corresponding author/s.

## Ethics Statement

The animal study was reviewed and approved by the Administration of Affairs Concerning Experimental Animals of China.

## Author Contributions

QF and ML designed the experiments and wrote the manuscript. RL designed the sparse labeling system. QF constructed, packaged, injected the AAV vectors, and reconstructed the neurons. SA, AL, and HG performed image and reconstructed data registration. SA, QF, and RW performed the quantitative analysis. All authors contributed to the article and approved the submitted version.

## Funding

This study was supported by the Beijing Municipal Government.

## Conflict of Interest

The authors declare that the research was conducted in the absence of any commercial or financial relationships that could be construed as a potential conflict of interest.

## Publisher's Note

All claims expressed in this article are solely those of the authors and do not necessarily represent those of their affiliated organizations, or those of the publisher, the editors and the reviewers. Any product that may be evaluated in this article, or claim that may be made by its manufacturer, is not guaranteed or endorsed by the publisher.

## Supplementary Material

Supplementary Figure 1 Schematics show the strategy of sparsely labeling neurons in the ventral pallidum (VP). **(A)** The paradigm of AAV vector infusions for sparse labeling of VP neurons. **(B)** The paradigm of AAV vector infusions for sparse labeling of VP neurons.

Supplementary Figure 2 Horizontal view of 41 fully reconstructed VP neurons.

Supplementary Table 1 Anatomical terminology.
