# Supplementary figures and images for: Whole-Brain Reconstruction of Neurons in the Ventral Pallidum Reveals Diverse Projection Patterns
Source: Front Neuroanat. 2021 Dec 16;15:801354. doi: 10.3389/fnana.2021.801354 (PMC8716739; doi:10.3389/fnana.2021.801354)

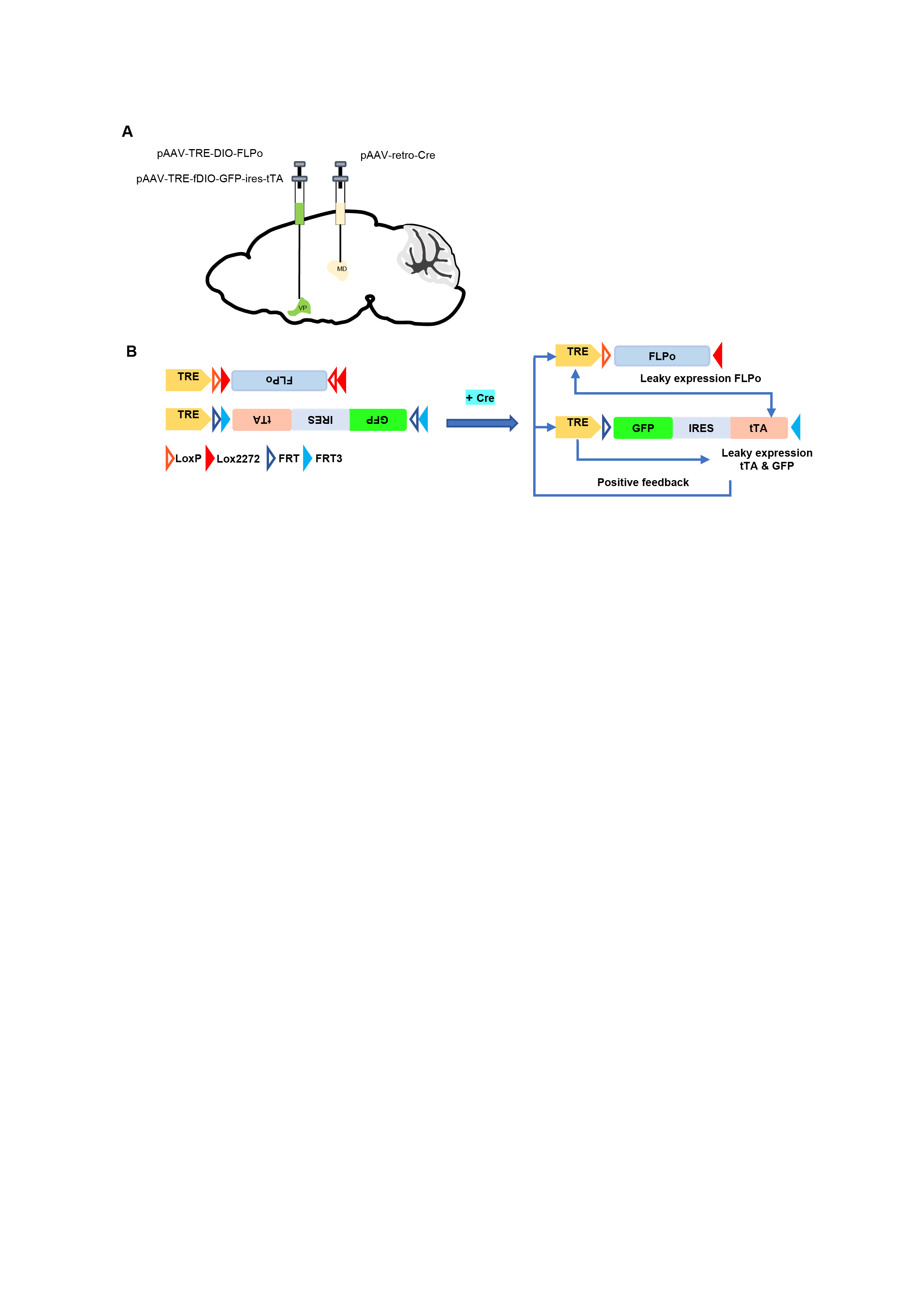

Supplement: Supplementary Figure 1 — Schematics show the strategy of sparsely labeling neurons in the ventral pallidum (VP). (A) The paradigm of AAV vector infusions for sparse labeling of VP neurons. (B) The paradigm of AAV vector infusions for sparse labeling of VP neurons. [file Image_1.JPEG]

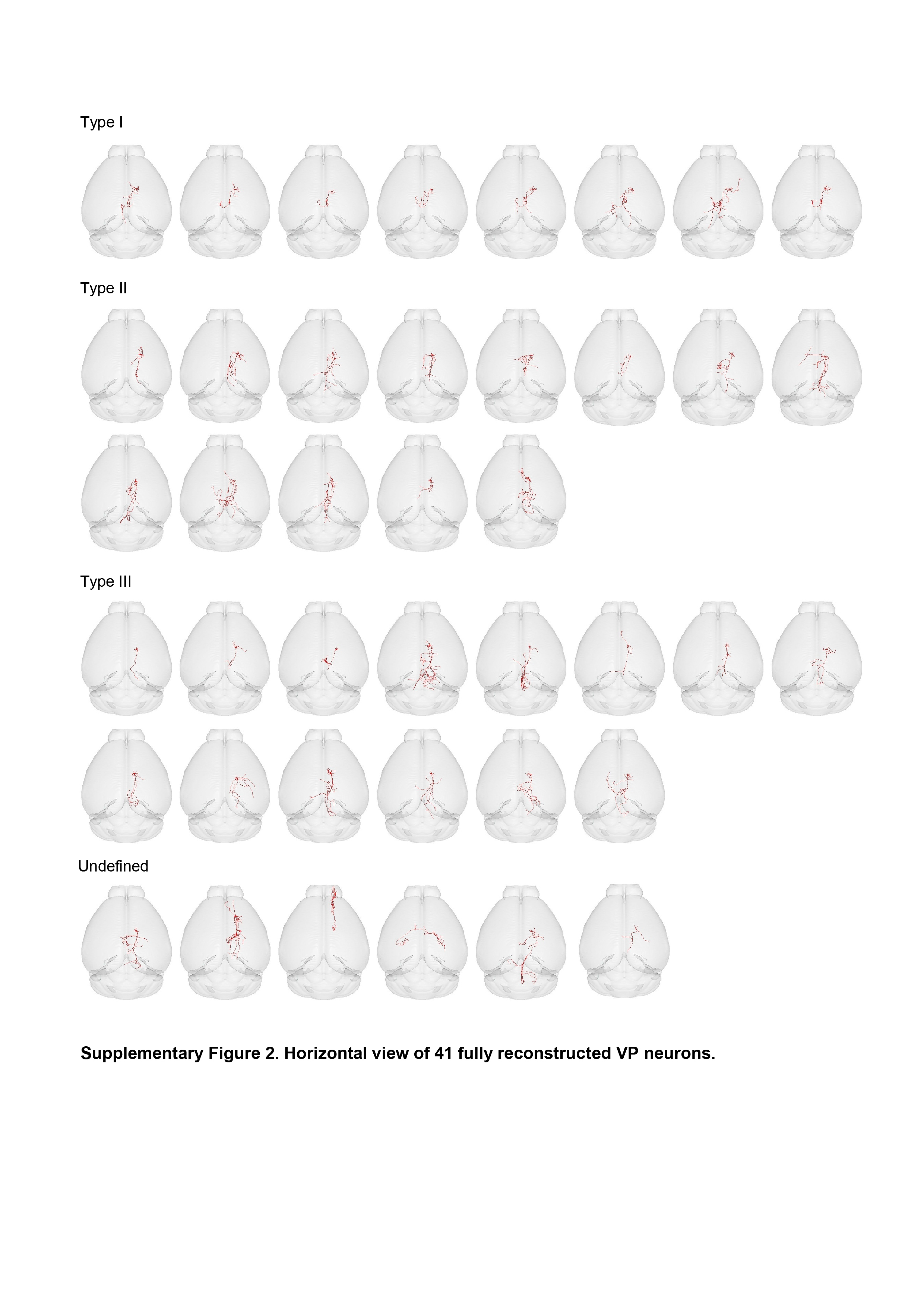

Supplement: Supplementary Figure 2 — Horizontal view of 41 fully reconstructed VP neurons. [file Image_2.JPEG]
