# Supplementary material for: Whole-Brain Reconstruction of Neurons in the Ventral Pallidum Reveals Diverse Projection Patterns
Source: Front Neuroanat. 2021 Dec 16;15:801354. doi: 10.3389/fnana.2021.801354 (PMC8716739; doi:10.3389/fnana.2021.801354)
Supplement: Supplementary Table 1 — Anatomical terminology. [file Data_Sheet_1.PDF]

Supplementary Table 1. Anatomical terminology

Abbreviation	Full anatomical terms of location
BMA	Basal Medial Amygdala
COA	Cortical amygdalar area
AAA	Anterior amygdalar area
CEA	Central amygdalar nucleus
MEA	Medial amygdalar area
CP	Caudoputamen
FS	Fundus of striatum
BST	Bednuclei of the stria terminalis
MA	Magnocellular nucleus
GPe	Globus pallidus, externa
GPi	Globus pallidus, interna
NDB	Diagonal band nucleus
SI	Substantia innominata
ATN	Anterior tegmental nucleus
RT	Reticular nucleus of the thalamus
VAL	Ventral anterior-lateral complex of the thalamus
MD	Mediodorsal nucleus of thalamus
LH	Lateral habenula
MH	Medial habenula
PVT	Paraventricular nucleus of the thalamus
CL	Central lateral nucleus of the thalamus
PF	Parafascicular nucleus
RE	Nucleus of reunions
SPF	Subparafascicular nucleus
SPA	Subparafascicular area
CM	Central medial nucleus of the thalamus
PCN	Paracentral nucleus
VP	Ventral posterior complex of the thalamus
VM	Ventral medial nucleus of the thalamus
LHA	Lateral hypothalamic area
LPO	Lateral preoptic area
PH	Posterior hypothalamic nucleus
MPO	Medial preoptic area
PST	Preparasubthalamic nucleus
PSTN	Parasubthalamic nucleus
SUM	Supramammilary nucleus
PVHd	Paraventricular hypothalamic nucleus, descending division
FF	Fields of Forel
AHN	Anterior hypothalamic nucleus
DMH	Dorsomedial nucleus of the hypothalamus
VLPO	Ventrolateral preoptic nucleus
MEV	Midbrain trigeminal nucleus
NPC	Nucleus of the posterior commissure
PPN	Pedunculopontine nucleus
SC	Suprerior colliculus
SNc	Substantia nigra, compact part
SNr	Substantia nigra, reticular part
RN	Red nucleus
MRN	Midbrain reticular nucleus
VTA	Ventral tegmental area
RR	Midbrain reticular nucleus, retrorubral area
PB	Parabrachial nucleus

PCG	Pontine central gray
PRNc	Pontine reticular nucleus, caudal part
PRNr	Pontine reticular nucleus
hbc	habenular commissure
ll	lateral lemniscus
sm	stria medullaris
scp	superior cerebelar peduncles
mlf	medial longitudinal fascicle
fr	fasciculus retroflexus
ml	medial lemniscus
